# Apparent overuse of antibiotics in the management of watery diarrhoea in children in Abakaliki, Nigeria

**DOI:** 10.1186/s12879-019-3899-1

**Published:** 2019-03-21

**Authors:** Akinwale M. Efunshile, Obumneme Ezeanosike, Chukwuemeka Chijioke Nwangwu, Brigitte König, Pikka Jokelainen, Lucy J. Robertson

**Affiliations:** 1 Department of Medical Microbiology, Ebonyi State University, Abakaliki, Nigeria; 2 Department of Medical Microbiology, Federal Teaching Hospital, Abakaliki, Nigeria; 3 Department of Paediatrics, Federal Teaching Hospital, Abakaliki, Nigeria; 4 Department of Paediatrics, Ebonyi State University, Abakaliki, Nigeria; 5 Institute of Medical Microbiology and Epidemiology of Infectious Diseases, University Teaching Hospital, Leipzig, Germany; 6 Laboratory of Parasitology, Department of Bacteria, Parasites & Fungi, Infectious Disease Preparedness, Statens Serum Institut, Copenhagen, Denmark; 7 Parasitology, Department of Food Safety and Infection Biology, Faculty of Veterinary Medicine, Norwegian University of Life Sciences, PO Box 369 Sentrum, 0102 Oslo, Norway

**Keywords:** Aetiology, Antibiotic treatment, Nigeria, Paediatric diarrhoea, Supportive therapy

## Abstract

**Background:**

Diarrhoea remains an important cause of childhood mortality in Nigeria, with *Rotavirus* and *Cryptosporidium* reported to have the highest contribution. However, high use of antibiotics for treatment of paediatric diarrhoea has been observed, although World Health Organization guidelines discourage the use of antibiotics for treating acute diarrhoea. Here we investigated more closely management and treatment practices for acute paediatric diarrhoea, both in home and healthcare settings.

**Methods:**

Children under 5 years of age (*n* = 199) presenting at healthcare centres in Abakaliki, Nigeria with acute watery diarrhoea were included in the study. Background information on the children was collected by questionnaire, including home treatments, and clinical information including symptoms and treatment were provided by the healthcare centres. Analysis of faecal samples from the children indicated that over 90% had *Rotavirus* infection and over 6% *Cryptosporidium* infection. Data were compiled in a spreadsheet and analysed for associations between variables and use of antibiotics using logistic regression analysis.

**Results:**

Although most children were treated supportively (oral rehydration solution and intravenous fluids at home and in healthcare settings, respectively) over 15% were given anti-diarrhoea drugs at home and over 85% were also prescribed antibiotics at the healthcare centre, mostly ciproflaxin, but also metronidazole and gentamycin. The only variable positively associated with antibiotic prescription was diarrhoea more than three times per 24 h at admission.

**Conclusions:**

It is clear that young children presenting with acute watery diarrhoea to healthcare centres in Abakaliki are likely to be prescribed antibiotics, despite there being no obvious reason that this treatment is appropriate. Our study results support the need for institution-based antimicrobial stewardship being implemented in Nigeria.

## Background

Despite the impressive reductions in mortality due to diarrhoea in recent years, diarrhoea remains an important childhood killer in many countries. In Nigeria, mortality among children below the age of 5 years from diarrhoea was 331.3 per 100,000 children in 2016, although there has been a reduction in mortality in this age group of over 20% between 2005 and 2015 [1, 2]. Among the aetiologies associated with mortality due to diarrhoea in Nigeria in children under 5 years, the mortality data assessment per pathogen conducted by the research group estimated that rotaviral enteritis has the highest impact (45%), with cryptosporidiosis in 2nd place considered responsible for 14.3% [1]; other diarrhoeal agents associated with deaths in this age group include *Adenovirus* (10.3%), *Shigella* (6.1%), *Salmonella* spp. (4.2%), *Norovirus* (3.6%), *Entamoeba histolytica* (2.6%), *Vibrio cholera* (3.5%), enteropathogenic *E. coli* (3.3%), and *Campylobacter* spp. (2.1%) [1]. Although Nigeria was not included in the Global Enteric Multicenter Study (GEMS) from which numerous articles have been published, these data have also indicated the importance of *Rotavirus* and *Cryptosporidium* as agents of diarrhoeal disease in young children in low income countries [3].

Despite the inaccuracies inherent in such estimates, it is clear that in Nigeria, as in many countries where mortality due to diarrhoeal disease is an important issue, the majority of these are not due to bacterial infections, but viral infections and some to protozoan infections. As the necessary resources for identification of diarrhoeal agents are often lacking in countries where they are most needed, the World Health Organization (WHO) has developed treatment guidelines for treating diarrhoea and aimed at healthcare workers [4]. Recommended practices consist of low-cost supportive interventions such as use of oral rehydration solution (ORS), intravenous fluids, breastfeeding, continuous feeding, zinc tablets [4] and community-based management practices by parents/guardians [5]. WHO guidelines explicitly discourage the use of antibiotics for treating acute diarrhoea as they will be of no effect in the majority of cases, due to non-bacterial aetiology [6]. Indeed, as noted by Bruzzese et al. (2018), antibiotic therapy is generally not necessary for treatment of acute diarrhoea in children [7]. Use of antibiotics for non-bacterial illnesses is considered widely as misuse of antibiotics; it tends to promote antimicrobial resistance, increase healthcare costs, and may sometimes even be associated with increased morbidity [6]. Thus, WHO guidelines indicate that the use of antimicrobial agents should be restricted to cases of bloody diarrhoea and cholera cases with severe dehydration. Risk factors indicating antibiotic therapy in children with acute diarrhoea have been also summarised to include age of below 3 (or 6) months and severe presentation – for both of which evidence is poor, but there are strong indications – and malnutrition and chronic underlying disease [7].

WHO does not currently have any guidelines or recommendations regarding use of probiotics in diarrhoea management, but in many healthcare settings, they are widely used as adjunct therapy. Although some studies have presented data that probiotic treatment reduces the duration and severity of diarrhoea [7–9], other studies have shown that probiotic treatment provided no benefits to patients in cases of childhood diarrhoea [10, 11].

In our study on some aetiologies of diarrhoea in Nigerian children under 5-years of age [12], we observed high use of antibiotics for treatment of diarrhoea both at home and in the healthcare setting. This observation is further investigated and discussed in this article.

## Methods

### Study design, setting, and study population

We carried out a study at two large health centres within Abakaliki, Nigeria; further details are provided elsewhere [11]. All children under 5-years of age with acute watery diarrhoea and without malaria, respiratory tract infection, or other diagnosed disease (exclusion criteria), who were admitted to the two healthcare centres during the study period were invited to participate in our study until the target sample size of 200 children was reach. The study period was from December 2016 to March 2017. Children were included in the study following informed consent by their parents or guardians. Information from the healthcare centres regarding symptoms and treatment were provided by the treating paediatricians using a number system such that it was not possible to identify the individual children, and the treatment records of each patient were also checked.

The children included in the study were followed up by telephone call to their homes/parents one month after discharge.

A description of the study population (demographic information, symptom information etc.) is included in the results.

### Ethical approval

Approval for the study was provided by the Review and Ethics Committee (REC) of the Federal Teaching Hospital, Abakaliki. An informed consent form was signed by parents/guardians of the children prior to enrolment in the study; information in the form made clear that participation in the study was voluntary and participants could opt out of the study at any time without prejudice to the quality of treatment received by their children. Questionnaire data were coded to be anonymous such that it is not possible to identify individual patients. Information was collected using the language that the caregivers felt most comfortable using.

### Data handling and statistical analysis

Data were initially compiled in an excel spreadsheet. Statistical analyses focused on variables potentially associated with use of antibiotics (dichotomous outcome, yes/no) for the diarrhoeal episodes. OpenEpi (www.openepi.com) was used to calculate 95% confidence intervals for proportions that received antibiotics (Mid-P Exact) and to evaluate associations using contingency Tables (2-tailed *P*-value, Mid-P Exact). Logistic regression analyses for identifying variables associated with using antibiotics were performed using Stata 13.1 (StataCorp, College Station, TX, USA). After univariable (crude) analyses, a multivariable model building was attempted, by including all variables followed by exclusion of those that were not significant and not confounders. *P* values < 0.05 were considered significant.

## Results

Most children included in the study were younger than 1 year (mean age = 10.4 months, median age = 9 months, age range = 1 to 48 months), and the majority were boys (61.4% male and 38.6% female; gender was not reported for 6 children). Background information on the children was collected using a questionnaire. The questionnaire covered socio-demographic factors, other symptoms observed, and management of the diarrhoeal episode. The sample included children from both rural (44.7%) and urban areas (55.3%) (for one child both options were reported; coded as missing data). *Rotavirus* infection was diagnosed in 92.1% (175/190) of the children and *Cryptosporidium* infection in 6.5% (13/199) of the children [9]. Bacteriological examination of the faecal samples was not performed. In addition to the diarrhoea, other concurrent symptoms were commonly reported including fever (> 98%), vomiting (> 92%), and mucus in stool (approximately 70%). Although for a few children parents/guardians reported blood in the stool (5.0%), this was not observed in the samples provided at the healthcare centres. “Rice-water diarrhoea” was also not observed. The duration of the diarrhoeal episode ranged from 2 to18 days (mean duration was 6.3 days and median 6 days). For around 25% of the children, diarrhoea had continued for more than a week, and the majority of the children (over 70%) had diarrhoeal episodes more than 3 times per 24 h upon admission.

Although the majority of children with watery diarrhoea enrolled in this study were treated with supportive treatment (ORS, intravenous fluids (IVF), zinc tablets, vitamin A), both in the domestic setting and the healthcare setting, a substantial majority, 86.9%, received antibiotics and over 30% received probiotics in the healthcare setting (Table 1).

**Table 1:** Management practices of children with watery diarrhoea, at home and in the healthcare setting

Settings	Number (%)
At home	
Continuous feeding	118 (59.0)
Non-prescription anti-diarrhoea drugs	31 (15.5)
Oral rehydration solution	196 (98.0)
In healthcare setting	
Intravenous fluid (IVF)	185 (92.5)
Antibiotics	173 (86.9)^a^
Probiotics^b^	73 (36.5)
Zinc tablets	196 (98.0)
Vitamin A	163 (81.9)

^a^Use of antibiotics was not reported for one child

^b^A commercial preparation (Floranorm) supplied by the hospital pharmacies in the study area and containing live non-pathogenic *Saccharomyces boulardii*

Regarding the antibiotics used, ciprofloxacin was used in most cases, for 72.4%, metronidazole for 30.2% and gentamycin for 15.1%. In some cases, antibiotic combinations were used, with ciprofloxacin and metronidazole used in combination in 22% of the children.

A comparison of the use of antibiotics according to different variables is shown in Table 2. Although for most variables investigated, there was no difference in use of antibiotics, the proportion of children receiving antibiotics was statistically significantly higher among children with diarrhoea more than three times per 24 h at admission than among with children with less frequent diarrhoea at admission (Table 2).

**Table 2:** Use of antibiotics for treatment of watery diarrhoea in children up to 4 years of age at two large health care centres in Abakaliki, Nigeria

Variable	N*	Treated with antibiotics Number (%)	95% confidence interval (mid-P Exact)	*P* value
Age < 1 year	136	117 (86.0)	79.4–91.1	0.598
Age 1–4 years	63	56 (88.9)	79.3–95.0	
Female	74	62 (83.8)	74.1–90.9	0.299
Male	119	106 (89.1)	82.5–93.8	
Urban	88	72 (81.8)	72.7–88.9	0.066
Rural	110	100 (90.9)	84.4–95.3	
No fever	3	3 (100)	36.8–100.0	0.656
Fever	196	170 (86.7)	81.4–91.0	
No vomiting	15	12 (80.0)	54.7–94.7	0.423
Vomiting	184	161 (87.5)	82.1–91.7	
No mucus in stool	59	54 (91.5)	82.2–96.8	0.247
Mucus in stool	137	117 (85.4)	78.7–90.6	
No blood in stool	189	163 (86.2)	80.8–90.6	0.238
Blood in stool	10	10 (100.0)	74.1–100.0	
Diarrhoea duration: < 1 week	148	129 (87.2)	81.0–91.9	0.854
Duration of diarrhoea: 1 week or more	51	44 (86.3)	74.7–93.8	
Diarrhoea frequency: up to 3 times per 24 h	58	45 (77.6)	65.6–86.9	0.018**
Diarrhoea frequency: > 3 times per 24 h	141	128 (90.8)	85.1–94.8	
Total	199	173 (86.9)	81.7–91.1	

*Whether antibiotics were used was not reported for one child, gender was not reported for six children, rural-urban was missing data for one child, and presence of mucus in stool was not reported for three children

**Statistically significant difference

The odds that antibiotics were used were 2.84 (95% CI 1.23–6.59) times higher for children that had experienced more than three episodes of diarrhoea during the 24 h prior to admission to the healthcare centre. Multivariable models were not yielded: all but this one variable were omitted from the model as non-significant.

Follow up of the children by telephone call one month after discharge revealed that one of the children had died 3 weeks after discharge and following apparent recovery from the diarrhoeic episode. Lack of autopsy information meant that it was not possible to ascertain the cause of death.

## Discussion

Our study demonstrated that of young children presenting to healthcare centres in Abakaliki, Nigeria with watery diarrhoea, the vast majority receive antibiotic treatment, despite there being no obvious diagnostic nor epidemiological basis that the children were suffering from infections for which this treatment is appropriate. Among the aetiologies associated with mortality due to diarrhoea in Nigeria in children under 5 years, rotaviral enteritis and cryptosporidiosis are the top two [1]. In the cohort investigated here, the majority had *Rotavirus* and other viral infections associated with diarrhoea (*Astrovirus*, *Adenovirus*), and around 6% had *Cryptosporidium* infections [12]; none of these agents would be affected by antibiotic treatment. Although the incidence of *Rotavirus* infection may seem unusually high, it should be noted that the study was conducted during the dry, cool months of the year that are particularly associated with *Rotavirus* infection [13].

It should be borne in mind that antibiotic treatment can be lifesaving for children with bacterial infections such as *Shigella* [14] and as bacteriological analyses were not performed for the children included in our study, we cannot exclude that some of the children also had bacterial infections and that these children benefitted from antibiotic treatment. We appreciate that the absence of testing for bacterial pathogens in these children is a limitation of our study, as this would have provided definitive information on whether antibiotic treatment could have resulted in any benefit. None of the children had “rice-water” diarrhoea (indicative of *Vibrio cholerae* infection). However, although none of the samples collected contained blood, for 10 children the parents reported blood in the stool, which may have suggested bacterial infection (particularly *Shigella*) to the prescribing clinicians. Although we are aware that infection with *Shigella* does not always present with bloody diarrhoea [15], it nevertheless seems likely that many of the children receiving antibiotics would have obtained no benefit from this treatment, and it may even have been detrimental, given the effects on the enteric immune system and gut microbiota [16]. It should be noted, however, that our sample consisted of children admitted to healthcare centres, and thus represents children with clinically severe diarrhoea. Indeed, almost all the children also had fever and vomiting, and the average duration of the diarrhoea was approximately 6 days. The common occurrence of fever could have been one reason why antibiotics were prescribed [7], as fever often accompanies bacterial infections such as shigellosis, campylobacteriosis, and salmonellosis. However, fever is also a common presentation in viral diarrhoea, particularly with *Rotavirus* [17, 18], and clinicians should be aware that these symptoms are insufficient to reach a conclusion of bacterial infection. The presence of fever has been reported to trigger inappropriate antibiotic prescription for watery diarrhoea by paediatric doctors in India [19, 20]. Furthermore, the use of an injectable drug cocktail, containing antimalarial, antibacterial and antiviral drugs, and referred to as “umbrella therapy” has been reported to be a common practice for treating severely ill febrile children in Sudan [21].

The duration of the diarrhoea may also have suggested to the prescribing clinicians that the child had a bacterial infection, as diarrhoea due to *Rotavirus* often ceases within a few days. However, this is not always the case, and diarrhoea due to protozoan infections may also be prolonged. Furthermore, we did not find an increased likelihood of antibiotics being prescribed if the duration of diarrhoea was longer than one week.

In addition, although the children were young (median age of 9 months), and age has been noted as a risk factor indicating antibiotic therapy [7], in our study older children were just as likely to be treated as younger ones.

Malnutrition is also a risk factor indicating antibiotic therapy in children with acute diarrhoea [7]. Although malnutrition was not specifically tested for in this study, it should be noted that the bi-directional relationship between malnutrition and diarrhoea has long been acknowledged [22]. As children presenting at hospital following a few episodes of diarrhoea will often appear malnourished, assessment of whether diarrhoea caused the malnutrition or vice versa remains a clinical challenge for paediatricians in this setting. It is possible that thoughts of malnutrition by the examining clinicians could have been a contributory reason for the high use of antibiotics in this study, however this was not explored and comments of malnutrition were not noted in the records. Studies from this region indicates that in healthy pre-school children the prevalence of malnutrition is around 5–10% [23], rising to around 20% in hospitalized children [24].

Of the variables we investigated, only high frequency of diarrhoea (over three times per 24 h) at admission was statistically significantly associated with the use of antibiotics. However, as over 77% of children that did not have such a high frequency of diarrhoeal episodes at admission also received antibiotics, it appeared that there was no clear clinical algorithm for the treatment decision. Whether fever was used as a potential indicator for using antibiotics [7] could not be investigated in this study, because almost all of the children had fever (Table 1). However, it could be worth noting that of the few children who did not present with fever all received antibiotic treatment (Table 1).

It should be noted that in this study, the relatively small sample size and the high overall use of antibiotics limited our ability to investigate which variables could have been interpreted by treating doctors as indicators for antibiotic treatment.

Although irrational inappropriate prescription of antibiotics by clinicians is a well-recognised global problem [6, 25], we consider that the rate of use (87%) in watery paediatric diarrhoea observed in this study to be astonishingly high and unacceptable, as it appeared that it was probably largely unreasoned. However, Nigeria is not alone; other studies from different African countries have shown similar results. In a Burkina Faso teaching hospital, febrile children under five-years of age and presenting with diarrhoea were treated with antibiotic in 90.9% of cases [26]; in Ethiopia, antibiotic prescription for childhood diarrhoea was reported to be over 86% [27], and around 80% in Tanzania [28]. Thus, use of antibiotics in Africa clearly merits more focus.

The reasons for inappropriate use of antibiotics generally result from the complex interactions between supply and demand, and involves both professionals and unqualified personnel in different sectors, including the retail sector and in healthcare [29]. A study among physicians in India indicated that less than 20% had good overall knowledge of diarrhoeal disease and its management, and that around only 17% prescribe antibiotics on the basis of appropriate clinical investigation [30]. A similar study in Iraq and Afghanistan also showed that only 30% of practitioners had good knowledge of diarrhoea management, and fewer than one-third had correct knowledge of causative agents of diarrhoea [31]. Furthermore, a recent study of antibiotic prescription patterns among Nigerian doctors showed that 97% prescribe antibiotics frequently and mostly without laboratory support, and that institutional policies or guidelines regarding antimicrobial therapy are generally lacking [32]. Clinicians may choose to prescribe antibiotics due to concerns about enteropathogenic bacteria such as *Campylobacter*, *Salmonella*, *Shigella* and enteropathogenic *E. coli*; however, they should be aware that these bacterial pathogens do not tend to be associated with acute watery diarrhoea in young children.

It is also clear that patients themselves (or the parents/guardians of paediatric patients) may expect, or even demand, antibiotic treatment [33], and this may put greater pressure on the clinician to prescribe a treatment that they themselves do not consider appropriate. In this respect, the socio-cultural rationale for antibiotic use in low-income settings may be an important driver that also needs to be taken into account [34]. The treatment expectations of parents of children with acute watery diarrhoea attending the healthcare centres would be worth investigating in different parts of Africa, including Abakiliki. Administration of probiotics, instead of antibiotics, may be one approach to fulfilling patient/caretaker expectations of treatment when antibiotic treatment is not appropriate, and is part of the probiotic/antibiotic debate [35]. Although our study does not provide any evidence that children treated with probiotics had any advantage over those who did not receive probiotics, a review of 63 studies, 56 of which included infants and young children, indicates that probiotics appear to be safe and have clear beneficial effects in shortening the duration and reducing stool frequency in acute infectious diarrhoea [8]. Furthermore, guidelines from the European Paediatric Society note that administration of *Saccharomyces boulardii* preparations was associated with reduced duration and severity of acute watery diarrhoea in European children, when used in combination with rehydration therapy [36].

The problem of irrational antibiotic use is not limited to developing countries; it is also an issue in the USA where it has been reported that up to 47 million inappropriate antibiotic prescriptions are written in doctors’ offices and emergency departments annually, for viral conditions that do not warrant antibiotic treatment [37]. In order to tackle the menace of antibiotic misuse in USA, the Centers for Disease Control and Prevention (CDC) has launched a programme called “National Action Plan for Combating Antibiotic-Resistant Bacteria” with the goal that all hospitals will establish antibiotic stewardship programmes to help reduce inappropriate antibiotic prescriptions by 20% by the year 2020. One of the core elements of the programme is continuous education of clinicians about antimicrobial resistance and optimal prescribing of antibiotics [38]. In our opinion, such a programme should be rolled out globally, but particularly in countries like Nigeria where it is clear that there is an acute problem with inappropriate over-prescription of antibiotics, at least in some healthcare centres.

Another section of our study, investigated the care of the children in the home setting, before admission to the healthcare centres. Although the high use of supportive therapies such as ORS is laudable, the use of anti-diarrhoeal drugs in over 15% of cases calls for concern. Use of these over-the-counter drugs (e.g., kaolin-pectin or anti-motility agents) for children has long been recognised as contraindicated due to their lack of benefit and increased risk of side effects, including ileus, drowsiness, and nausea. In Ghana, a 4-year initiative (Strengthening Health Outcomes through the Private Sector (SHOPS) project) sought to decrease inappropriate use of such treatments (and antibiotics) for childhood diarrhoea using a number of strategies including training, support, and media campaigns [39]. Although antibiotic use declined markedly during the project, at follow-up it remained high, especially in cases of non-bloody diarrhoea. However, there was a lasting impact on using ORS and zinc, and it was suggested that a similar package of interventions has the potential to achieve rapid scale-up of use of ORS and zinc in other settings [39].

## Conclusions

The results of our study indicate that there is a problem with treatment of watery paediatric diarrhoea in Abakaliki, which we assume is probably replicated in other regions across Nigeria and Africa. The use of anti-diarrhoea treatments in the home should be urgently addressed, and so should the inappropriate use of antibiotics when children are admitted to healthcare centres. A nationwide survey of antibiotic use in Nigeria would provide us with a better understanding of the magnitude of the problem. We believe that a multi-pronged approach, targeting parents, caregivers, and physicians, is imperative to bring this situation under control. Physicians must be encouraged to consider not only bacterial diseases, in their differential diagnoses – but also infections with viruses and parasites. An approach similar to that of CDC in USA, which emphasizes institution-based antimicrobial stewardship, with emphasis on continuous medical education for clinicians on rational antibiotic use, would seem an appropriate place to start.

## Abbreviations

CDC: Centers for Disease Control and Prevention
CI: Confidence interval
GEMS: Global enteric multicenter study
IVF: intravenous fluid
ORS: Oral rehydration solution
REC: Review and ethics committee
SHOPS: Strengthening health outcomes through the private sector
USA: United States of America
WHO: World Health Organization
